# Case of Hermaphrodism, Involving the Operation of Castration, and Illustrating a New Principle in Juridical Medicine

**Published:** 1853-03

**Authors:** 


*Case of Hermaphrodism, involving the Operation of Castration, and illustrating a new principle in Juridical Medicine.* By S. D. Gross, M. D., Professor of Surgery in the Medical Department of the University of Louisville.

The following case, which came under my observation in 1849, will, if I mistake not, prove both novel and interesting to my professional brethren. So far as my information extends, there is no account of any operation for a similar object upon record.

The subject of the case, at the time I first saw her, was three years of age, having been born the 10th of July, 1846. She had always, up to this period, been regarded as a girl, and had been so pronounced at her birth by the accoucheur. At the age of two, however, she began to evince the tastes, disposition, and feelings of the other sex; she rejected dolls and similar articles of amusement, and became fond of boyish sports. She was well-grown, perfectly healthy, and quite fleshy. Her hair was dark and long, the eyes black, and the whole expression most agreeable. A careful examination of the external genitals disclosed the following circumstances: There was neither a penis nor a vagina; but, instead of the former, there was a small clitoris, and, instead of the latter, a superficial depression, or *cul-de-sac*, covered with mucous membrane, and devoid of every thing like an aperture, or inlet. The urethra occupied the usual situation, and appeared to be entirely natural; the nymphæ were remarkably diminutive; but the labia were well developed, and contained each a well-formed testis, quite as large and consistent as this organ generally is at the same age in boys. Her hips and chest, thighs and superior extremities, were perfect.

It being apparent, from the facts of the case, that it was one of malformation of the genital organs usually denominated hermaphrodism, the question occurred whether any thing could or ought to be done to deprive the poor child of that portion of the genital apparatus which, if permitted to remain until the age of puberty, would be sure to be followed by sexual desire, and which might thus conduce to the establishment of a matrimonial connection. Such an alliance, it was evident, could eventuate only in chagrin and disappointment, if not in disgrace, ruin of character, or even loss of life. Certainly, impregnation could never occur, and even copulation could not be performed, except in the most imperfect manner.

I need not say that I gave the subject all the consideration and reflection that I was capable of bestowing upon it. I was deeply sensible of the responsibility of my position. A new question, involving the rights and happiness of my little patient, and the dearest interests of her parents, was presented to me. I examined the case in all its bearings and relations—moral, physiological, and juridical; I appealed to the records of my profession for a precedent, and I sought the counsel of medical friends. The parents were anxious for an operation; they were intelligent, kind, and tender-hearted, and were willing to sacrifice every thing for the welfare of their child. Their only object was to save it from future suffering and misfortune. My own mind was made up; but, before I proceeded to take any further steps, I determined to consult my excellent friend and colleague, Professor Miller, in whose judgment and integrity every one who knows him has the utmost confidence. He saw the child and examined her. He viewed the case, as I had previously, in every possible aspect, and his conclusion was, that excision of the testes was not only justifiable, but eminently proper under the circumstances; that it would be an act of kindness and of humanity to the poor child, standing as she did toward society in the relation not of a boy or a girl, but of a neuter, to deprive her of an appendage of so useless a nature; one which might, if allowed to proceed in its development, ultimately lead to the ruin of her character and peace of mind.

Backed by such authority, I no longer hesitated what course to pursue. I performed the operation of castration on the 20th of July, 1849, aided by my pupils, Dr. D. D. Thompson, of this city, Dr. Greenburg R. Henry, of Burlington, Iowa, and Dr. William H. Cobb, formerly of Louisville, now of Cincinnati. The little patient being put under the influence of chloroform, I made a perpendicular incision, about two inches in length, into each labium down to the testis, which was then carefully separated from the surrounding structures, and detached by dividing the lower part of the spermatic cord. The arteries of the cord being secured with ligatures, the edges of the wound were brought together with twisted sutures, and the child put to bed. Hardly any blood was lost during the operation. About two hours after, the left labium became greatly distended and discolored; and, upon removing the sutures, the source of the mischief was found to be a small artery, which was immediately drawn out and tied. No unpleasant symptom of any kind ensued after this, and in a week the little patient was able to be up, being quite well and happy.

The testes were carefully examined after removal, and were found to be perfectly formed in every respect. The spermatic cords were natural.

I have seen this child repeatedly since the operation, as her parents live only a few squares from my office, and have carefully watched her mental and physical development. Her disposition and habits have materially changed, and are now those of a girl; she takes great delight in sewing and housework, and she no longer indulges in riding sticks, and other boyish exercises. Her person is well developed, and her mind uncommonly active for a child of her years.

I would fain present this example as a precedent in similar cases. The reasons which induced me to recommend and perform this operation, in the instance before me, have been already mentioned, and now, after a lapse of three years, I have no cause to regret the undertaking, or to think that I acted harshly and inconsiderately. If the records of surgery and medical jurisprudence are silent upon the subject; if the learned doctors of the Sorbonne, the fathers of the Royal Academy of Paris, and the Fellows of the Royal College of London have left us no precepts; and if the experience of the present day furnishes no examples; all this, and much more, does not prove that the practice here recommended is not perfectly just and proper, and vindicated upon every principle of science and humanity.

A defective organization of the external genitals is one of the most dreadful misfortunes that can possibly befall any human being. There is nothing that exerts so baneful an influence over his moral and social feelings, which carries with it such a sense of self-abasement and mental degradation, or which so thoroughly "maketh the heart sick," as the conviction of such an individual that he is forever debarred from the joys and pleasures of married life, an outcast from society, hated and despised, and reviled and persecuted by the world. Nothing but the most perfect resignation, and a well-founded confidence in the mercy and justice of the Creator, can render the lot of such a being at all supportable.

The subject of doubtful sex is one which has always, in all ages, and in all civilized countries, excited the warmest attention of the physiologist, the philosopher, and the medical jurist. Under the vague and ill-chosen name of hermaphrodism, invented at an early period of the world, was described every imaginable form of malformation of the genital and urinary organs, most dissimilar in character; and, consequently, were calculated to mystify and mislead the public mind. A class of beings was imagined, combining, it was said, the qualities of the male and female in the same individual, and capable of performing, within itself, the generative functions. The idea that such a union might exist, had its origin, no doubt, in fable. The reader of mythology need not be reminded here of the story of Hermaphroditus and the nymph Salmacis; how the former so ungallantly resisted the charms and entreaties of the latter, and how, finally, through the interposition of the gods, their bodies were united into one. The ignorance of medical men, the conceit and folly of legislators, and the mercenary conduct of many of the subjects of this variety of congenital malformation, served afterward, in no small degree, to perpetuate the error thus engendered, and to transmit it, in nearly all its ancient force, down to a comparatively recent period. Modern researches had done much to dissipate these absurdities, when the publication, in 1836, of the great work of Mons. Isidore St. Hilaire, entitled *Histoire des Anomalies de l' Organization*, set the long agitated question forever at rest, by demonstrating, in the most undeniable and conclusive manner, that there is no such thing as hermaphrodism, in the vulgar acceptation of the term; or, in other and more philosophical language, that the union of perfect male and female organs in one and the same individual, is an anatomical and physiological impossibility.

Much prejudice, leading often to the most cruel persecution, existed against this class of individuals among some of the nations of antiquity. The Athenians had a law, providing that all hermaphroditic children should be consigned to the flame; while the Romans ordained that they should be boxed up and thrown into the sea. In more recent times, all individuals of this description were excluded from holy orders, and from the office of judges, "because they were ranked with infamous persons, to whom the gates of dignity should not be opened." * Much of this prejudice has, fortunately, disappeared, under the benign influences of Christianity and civilization; but much still remains, and must continue in operation, as long as the human mind retains its present organization. If hermaphrodites are no longer burnt and drowned, stoned and persecuted, and mocked and reviled, they are universally regarded with a degree of prejudice, amounting, generally, to positive aversion; and as unfit for any offices of dignity, divine, legal, or political. If such be the fact, and no one can doubt it, every suggestion, calculated to ameliorate the condition of this unfortunate class of beings, by depriving them of their only incentives to matrimony, and thereby dooming them to everlasting celibacy, should be hailed as a valuable contribution to the science and humanity of the present age.—*Am. Jour. Med. Sciences.*

* Beck's Medical Jurisprudence, vol. i. p. 106, fifth ed. 1835.

